# Commentary: *Borrelia miyamotoi*: 43 Cases Diagnosed in France by Real-Time PCR in Patients With Persistent Polymorphic Signs and Symptoms

**DOI:** 10.3389/fmed.2020.00474

**Published:** 2020-09-02

**Authors:** Alex Wagemakers, Hein Sprong, Alexander Platonov, Joppe W. Hovius

**Affiliations:** ^1^Department of Medical Microbiology and Infection Prevention, Amsterdam UMC, Amsterdam, Netherlands; ^2^Centre for Infectious Disease Control, National Institute for Public Health and the Environment, Bilthoven, Netherlands; ^3^Central Research Institute of Epidemiology, Moscow, Russia; ^4^Section of Infectious Diseases, Department of Internal Medicine, Amsterdam UMC, Amsterdam, Netherlands

**Keywords:** *Borrelia miyamotoi*, PCR, non-specific symptoms, commentary, contamination, false-positive

## Introduction

*Ixodes* ticks are the vector of the *Borrelia burgdorferi* sensu lato complex causing Lyme borreliosis (LB) and of *Borrelia miyamotoi*, a relapsing fever *Borrelia* species causing *Borrelia miyamotoi* disease (BMD). The latter disease entity was first described in 2011 (1), and its clinical symptoms in patients in Asia, Europe, and the USA mostly consist of a flu-like illness (2). The recent Frontiers in Medicine article by Michel Franck et al. claims to have detected *Borrelia miyamotoi* DNA in 43 out of 824 French patients with a complex of non-specific symptoms lasting at least 6 months (3). However, we have serious doubts about the author's findings and conclusions. In this commentary, we describe evident shortcomings of this study and urge for a reconsideration of its interpretation and conclusions.

## Patient Description

The paper describes a poorly characterized patient population: it is unclear to which institutions they presented and how they were included in this study. Furthermore, the inclusion criteria are not described. Blood was collected, but it is unclear when and where this was done and how these samples were processed. Finally, while clinical characteristics of 31 patients with positive *B. miyamotoi* PCR and available questionnaires were described, the authors omitted to describe the clinical characteristics of PCR-negative patients and controls.

## PCR Method

The PCR that was performed was based on a single target (*glpq*), which was also present in the positive control and thus posing a risk for contamination, despite the necessary countermeasures and controls. Furthermore, the low number of negative healthy controls does not exclude the possibility of false-positives dominating the results in the studied patient group: the proportion of positive PCR findings in the patient group does not differ significantly from the healthy control group (*p* = 0.63, Fisher's exact test). Moreover, the median bacterial load described by Franck et al. was supposedly twenty times higher than in well-described patients with severe acute BMD (4). Thus, the PCR results presented in this paper appear to be at risk of representing contamination with either positive control or PCR amplicons. One obvious way to lower this risk would have been a second PCR targeting an independent target.

## Sequencing Results

**Figure 1:**
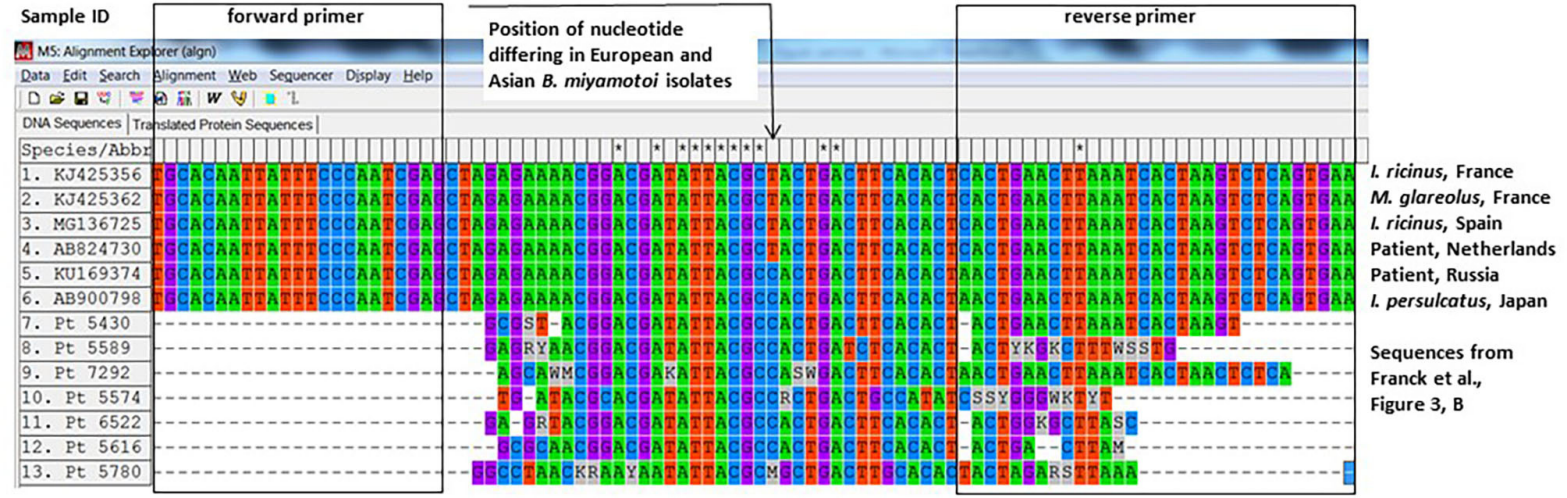
Alignment of *glpq* sequences described in Franck et al. with previously described sequences from West-European and Asian *Borrelia miyamotoi* isolates.

Another way to demonstrate that the positive PCRs were not false-positives is sequencing. The authors sequenced eight out of 32 positive samples and performed sequencing on the same fragment that was used in the qPCR. The authors used a plasmid control as a positive control in their qPCR assays, which contains a 94-bp fragment of the *glpq* gene from a Japanese *B. miyamotoi* isolate (HT-31, AB900798). This small and conserved fragment is 40 bp long (minus the primers) and differs from the Western-European *B. miyamotoi* isolates in one nucleotide (position 26, Figure 1). As far as we know, all Asian (*I. persulcatus*-associated) isolates contain a Cytosine whereas all known West-European (*I. ricinus*-associated) isolates contain a Thymidine at that position (Figure 1). Also, 12 French *B. miyamotoi* isolates (GenBank accession numbers KJ425352–KJ425363) from an independent study (5) contain a Thymidine at position 26, two of which are depicted in Figure 1. Six from seven of the *B. miyamotoi* sequences from the French patients in the study of Michel Franck et al. contained a Cytosine at position 26, identical to their positive control and deviant from all known *glpQ* sequences in European (*I. ricinus*-associated) *B. miyamotoi* isolates (Figure 1). It is therefore likely that the authors have amplified their positive control as a contaminant in these patient samples. Our request to obtain materials to perform an independent PCR was denied with the argument that blood samples and even DNA extracts were no longer available.

## Inconsistency of the Results With Previous Studies

The presented results appear to be in conflict with current knowledge on *B. miyamotoi* pathogenesis and disease manifestations: The patients included in this study had symptoms for at least 6 months, and out of 31 patients with a questionnaire available, 35 percent described relapsing fevers. It is unclear what exact pattern these patients described, how high the fevers were, how long this lasted, and whether other diagnoses were identified. Furthermore, in studies with PCR-positive well-described BMD patients, relapsing fever has only been described as a rare and temporary phenomenon limited by either the use of antibiotics or by time (not more than a couple of weeks) (1, 6).

## Discussion

Currently, the diagnosis of Lyme borreliosis but also other tick-borne diseases suffers from the poor diagnostic yield of serology during the early disease manifestations and the lack of sensitivity of PCR on blood and CSF. Although clinical diagnosis can indeed be very difficult, this has also created a large gray area and symptoms unrelated to LB have been attributed to the disease. The above has resulted in discontent within the public domain, both under- and over diagnoses, delay of proper therapy, and alleged failures of therapy. In contrast, for *B. miyamotoi* disease, the disease manifestations are thus far clearly defined, and PCR on blood appears to be a reliable tool to diagnose active infection. We have here outlined why the recent study by Franck et al., supposedly showing that long-lasting non-specific symptoms are associated with active *B. miyamotoi* infections, has too many shortcomings to redefine the clinical symptoms of BMD. In our opinion, their findings and conclusions should not have any implications for clinical decision-making.

## Author Contributions

AW, HS, AP, and JH have written the manuscript. HS and AP have performed the gene alignment, AP has generated the figure. All authors contributed to the article and approved the submitted version.

## Conflict of Interest

The authors declare that the research was conducted in the absence of any commercial or financial relationships that could be construed as a potential conflict of interest.
